# A multicentre, randomised, controlled trial to assess the safety, ease of use, and reliability of hyaluronic acid/carboxymethylcellulose powder adhesion barrier versus no barrier in colorectal laparoscopic surgery

**DOI:** 10.1186/1745-6215-15-413

**Published:** 2014-10-27

**Authors:** Stéphane V Berdah, Christophe Mariette, Christine Denet, Yves Panis, Christophe Laurent, Eddy Cotte, Nöel Huten, Eliane Le Peillet Feuillet, Jean-Jacques Duron

**Affiliations:** Chirurgie Digestive, Hôpital Nord, CERC (Centre d’Enseignement et de Recherche Chirurgical), Aix-Marseille Université, Chemin des Bourrellys, 13915 Marseille, Cedex 20, France; Chirurgie Digestive et Générale, Hôpital Claude Huriez, Centre Hospitalier Régional Universitaire, 2 Avenue Oscar Lambret, 59037 Lille, Cedex, France; Institut Mutualiste Montsouris, 42 Boulevard Jourdan, 75674 Paris, Cedex 14, France; Chirurgie Colorectale, Hôpital Beaujon, 100 Boulevard du Général Leclerc, 92110 Clichy, France; Chirurgie Générale et Digestive, Hôpital Saint André, 1 Rue Jean Burguet, 33000 Bordeaux, France; Chirurgie Digestive et Endocrinienne, Centre Hospitalier Lyon-Sud, Chemin du Grand Revoyet, 69310 Pierre-Bénite, France; Centre Hospitalier Régional Universitaire de Tours, Avenue de la République, 37170 Chambray-lès-Tours, France; Sanofi (Genzyme), 33-35 Boulevard de la Paix, 78105 Saint-Germain-en-Laye, Cedex, France; Chirurgie Générale et Digestive, Hôpital Pitié Salpêtrière, 47-83 Boulevard de l’Hôpital, 75013 Paris, France

**Keywords:** Adhesion barrier, Colorectal surgery, Laparoscopy, Surgical adhesions, Surgical site infection

## Abstract

**Background:**

Intra-peritoneal adhesions are frequent following abdominal surgery and are the most common cause of small bowel obstructions. A hyaluronic acid/carboxymethylcellulose (HA/CMC) film adhesion barrier has been shown to reduce adhesion formation in abdominal surgery. An HA/CMC powder formulation was developed for application during laparoscopic procedures.

**Methods:**

This was an exploratory, prospective, randomised, single-blind, parallel-group, Phase IIIb, multicentre study conducted at 15 hospitals in France to assess the safety of HA/CMC powder versus no adhesion barrier following laparoscopic colorectal surgery. Subjects ≥18 years of age who were scheduled for colorectal laparoscopy (Mangram contamination class I‒III) within 8 weeks of selection were eligible, regardless of aetiology. Participants were randomised 1:1 to the HA/CMC powder or no adhesion barrier group using a centralised randomisation list. Patients assigned to HA/CMC powder received a single application of 1 to 10 g on adhesion-prone areas. In the no adhesion barrier group, no adhesion barrier or placebo was applied. The primary safety assessments were the incidence of adverse events, serious adverse events, and surgical site infections (SSIs) for 30 days following surgery. Between-group comparisons were made using Fisher’s exact test.

**Results:**

Of those randomised to the HA/CMC powder (n = 105) or no adhesion barrier (n = 104) groups, one patient in each group discontinued prior to the study end (one death in each group). Adverse events were more frequent in the HA/CMC powder group versus the no adhesion barrier group (63% vs. 39%; *P* <0.001), as were serious adverse events (28% vs. 11%; *P* <0.001). There were no statistically significant differences between the HA/CMC powder group and the no adhesion barrier group in SSIs (21% vs. 14%; *P* = 0.216) and serious SSIs (12% vs. 9%; *P* = 0.38), or in the most frequent serious SSIs of pelvic abscess (5% and 2%; significance not tested), anastomotic fistula (3% and 4%), and peritonitis (2% and 3%).

**Conclusions:**

This exploratory study found significantly higher rates of adverse events and serious adverse events in the HA/CMC powder group compared with the no adhesion barrier group in laparoscopic colorectal resection.

**Trial registration:**

ClinicalTrials.gov NCT00813397. Registered 19 December 2008.

## Background

Intra-peritoneal adhesions are estimated to occur after 93% to 100% of upper abdominal laparotomies and after 67% to 93% of lower abdominal laparotomies [1]. Adhesions form as a result of surgical trauma or infection/inflammation, and comprise fibrous scar tissue that abnormally connects tissues and organs [2]. They are the most common cause of small bowel obstructions [3–6] and are associated with infertility and possibly chronic pain. Adhesions may also prolong operating time in subsequent surgery, and cause complications such as unintentional enterotomy [2, 6–8].

There is no effective treatment for adhesions, and surgery to deal with the consequences of adhesions, such as small bowel obstruction, often results in further adhesion formation [1, 2]. Adhesion prevention should therefore be considered the best management strategy [2, 9], although this is not widely demonstrated in the literature. A good surgical technique (e.g., minimal tissue trauma, avoiding introduction of foreign materials) can reduce adhesion formation, but is not sufficient for prevention [2, 9]. Adhesions may be reduced by using laparoscopy versus open surgery [10–12], which results in a reduction in adhesion-related complications, such as small bowel obstruction, but does not totally prevent adhesion formation [6]. A number of anti-adhesion products are available for adjuvant use during surgery, with various formulations including films, fabrics, gels, and fluids. These products act as a temporary mechanical barrier to separate organs and tissues for a short time while healing takes place. Despite the number of agents available worldwide, few have demonstrated efficacy in reducing post-surgical adhesions, and limited conclusions can be drawn on the effect of reducing adhesions, as no agent has been shown to improve the myriad negative outcomes commonly associated with adhesions [13].

In abdominal surgery, a hyaluronic acid/carboxymethylcellulose (HA/CMC) film adhesion barrier has been shown to reduce adhesion formation with a favourable safety profile [13–17]. A powder formulation of HA/CMC (HA/CMC powder; Sepraspray™ Adhesion Barrier, Genzyme Corp., Cambridge, MA, USA) was developed for application during laparoscopic procedures. Preclinical animal models indicated that this formulation was effective in reducing adhesion formation and did not disrupt normal wound healing [18, 19]. In a randomised pilot study in women undergoing laparoscopic myomectomy, there was a trend towards a reduction in adhesions with HA/CMC powder versus no adhesion barrier, with a favourable safety profile [20]. The primary objective of this study was to assess the safety of HA/CMC powder versus no adhesion barrier following laparoscopic colorectal and/or small bowel surgery (high risk for morbidity [21]), as determined by the incidence of adverse events, serious adverse events, superficial surgical site infections (SSIs) and deep SSIs, such as fistula, sepsis, abscess, and peritonitis.

## Methods

This was an exploratory, prospective, randomised, single-blind, parallel-group, Phase IIIb, multicentre study conducted at 15 hospitals in France (ClinicalTrials.gov Identifier: NCT00813397). The study comprised a selection visit 1 to 56 days prior to the planned surgery (Day 0) and two postoperative follow-up assessments (day of discharge or 7 ± 3 days post-surgery, and end-of-study assessment 28 to 35 days post-surgery). This follow-up duration was based on postoperative guidelines on infections [22] and the knowledge that HA/CMC is resorbed from the peritoneal cavity within 7 days and fully eliminated from the body in <28 days [23].

The study was carried out in compliance with the Declaration of Helsinki and the principles of the French Good Clinical Practice regulations/clinical research guidelines. The protocol and patient consent forms were approved by an independent ethics committee (reference number 208 R09, Sud-Méditerranée II, Marseille, France). All patients signed an informed consent form. An independent review committee of four independent experts was established to provide real-time expert review of safety reports and assess all safety data at study end.

### Participants

Men and women ≥18 years of age were eligible if they were scheduled to undergo a laparoscopic colorectal and/or small intestine surgical resection of Mangram contamination class I, II, or III [22] within 8 weeks of selection, whatever the aetiology (including cancer). Participants were also required to have an American Society of Anesthesiologists Physical Status Classification of P1, P2, or P3 and women of childbearing potential were required to use an effective contraceptive method for 1 month after randomisation. The principal exclusion criteria were cancer requiring chemotherapy and/or radiotherapy within 30 days prior to or after surgery; current abdominal abscess and/or peritonitis; pregnancy; clinically significant cardiovascular, hepatic, neurologic, psychiatric, endocrine, or other major systemic disease that would interfere with the study or jeopardize patient outcomes within 30 days; and treatment with heparinic anticoagulants (except prophylaxis for deep vein thrombosis).

Additional exclusion criteria applied at the time of surgery were use of another medical device that may interfere with the study (e.g., prosthetic stitch, biological adhesive, haemostatic compress, surgical membrane, or physical barrier to prevent adherence); infection discovered in the abdominal cavity; change to Mangram contamination class IV; and conversion to laparotomy (although mini laparotomy was permitted, based on standard laparoscopic approaches in colorectal surgery).

### Treatment

Participants were randomised to the HA/CMC powder or no adhesion barrier group during the planned surgical procedure (Day 0) in a 1:1 ratio using a centralised randomisation list and an automated Interactive Voice Response System, following re-evaluation of their eligibility. Patients were blinded to their randomisation group throughout the study.

Patients assigned to HA/CMC powder received a single application to adhesion-prone areas but not to anastomoses or sutures. HA/CMC powder was applied with a single-use applicator attached to a sprayer to allow precise application to the required sites while minimizing potential dispersion to other sites. The amount applied was at the surgeon’s judgement, and ranged from 1 to 10 g, with the maximum determined based on a previous study of HA/CMC film [24]. In patients assigned to the no adhesion barrier group, no adhesion barrier or placebo was applied. In both groups, closure of trocar sites was performed according to the normal routine of the surgeon; peritoneal closure was not performed.

### Safety assessments

The primary objective of this study was to compare the incidence of adverse events, serious adverse events, superficial (incisional) SSIs, and deep SSIs in the HA/CMC powder and no adhesion barrier groups for 30 days following surgery. Any suspected intra-abdominal abscess was investigated by CT-scan/MRI. Any suspicion of sepsis was confirmed by at least one positive blood culture and specific symptoms. Any suspicion of peritonitis was confirmed by positive bacterial cultures from peritoneal swabs of drainage during interventional radiology or surgery. All such SSIs were followed up until resolution. In the HA/CMC powder group, the relationship of adverse events to treatment was judged by investigators as not related, unlikely, possibly, probably, or definitely related. Events that were routinely observed during the postoperative period (e.g., pain, nausea, vomiting) were not reported as an adverse event unless they occurred with unusual severity according to the investigator’s judgement, or required unusual or specific management.

The duration of hospitalisation after surgery was noted along with intraoperative parameters and perioperative parameters. Exposure to HA/CMC powder was determined from the amount of powder applied (grams) and duration of application (minutes).

### Ease of use, manageability, and reliability assessments

Ease of use was evaluated by the surgeon using a 4-point scale (1 = very difficult, 2 = difficult, 3 = easy, 4 = very easy). HA/CMC powder was considered manageable if this item was scored 3 or 4. The ease of attaching the applicator to the sprayer and introducing the HA/CMC powder into the sprayer was assessed by the nurse in charge of surgical instruments in the operating room using the same 4-point scale. HA/CMC powder was considered manageable if both items were scored 3 or 4. Reliability was assessed by the surgeon based on the ability to cover all target areas, apply a homogeneous layer, and deliver the suitable amount of HA/CMC powder using a 4-point scale (1 = very bad, 2 = bad, 3 = good, 4 = very good), with HA/CMC powder being considered reliable if each item was scored 3 or 4.

### Statistical analysis

As the study was exploratory, no sample size calculation was performed. Safety analyses were performed on the safety population, comprising all randomised patients who underwent surgery. Surgical and ease of use analyses were performed on the intent-to-treat population, described as all randomised patients who received treatment during surgery as determined by randomisation.

Safety data are reported in summary tables and between-group comparisons made using Fisher’s exact test. Descriptive statistics are provided for ease of use, manageability, and reliability.

A post-hoc analysis of risk factors for deep SSIs and serious adverse events was carried out. Logistic regression was performed for each covariate. The univariate model included treatment effect, covariate effect, and the effect for the interaction between treatment and the covariate. Covariates included gender, age, body mass index, smoking status, smoking frequency, use of drains, adhesiolysis, and type of anastomosis, among others. A significance level of 0.10 (deep SSIs) or 0.20 (serious adverse events) was used to select the individual covariates to be included in the final multivariate model for the backwards stepwise logistic regression analysis. The odds ratio (OR) and 95% confidence intervals (CI) were calculated.

### Role of the sponsor

The study sponsor was involved in the design of the study. Data were collected by investigators at each site. Patient randomisation and data management were carried out by a research organisation contracted by the study sponsor. Together with the study investigators, the sponsor participated in the analysis and interpretation of data, the writing of the manuscript, and the decision to submit the manuscript for publication.

## Results

**Figure 1:**
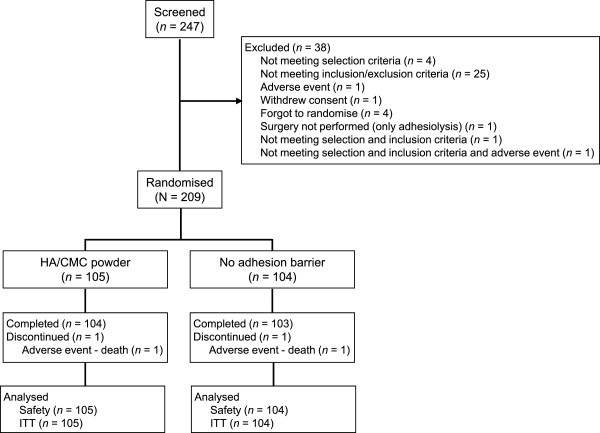
**Patient disposition.** ITT, intent-to-treat.

The study was carried out between September 2008 and July 2009. Patient disposition is summarized in Figure 1. Of 209 patients randomised to the HA/CMC powder (n = 105) or the no adhesion barrier (n = 104) groups, only one patient in each group discontinued prior to study end (one death in each group). Patient characteristics and medical history were similar (Table 1), except for a lower frequency of patients with concomitant allergic diseases (*P* = 0.031) and a higher frequency of patients taking concomitant penicillin combinations (significance not tested) in the HA/CMC powder group versus no adhesion barrier. Previous or concomitant immunosuppressive drugs were taken by 7/105 (7%) patients in the HA/CMC powder group and 12/104 (12%) patients in the no adhesion barrier group (*P* = 0.221), and corticosteroids by 15/105 (14%) and 22/104 (21%) patients, respectively (*P* = 0.193). Within the year prior to study randomisation (but not within 30 days before surgery), 10/105 (19%) of patients in the HA/CMC powder group and 7/104 (15%) in the no adhesion barrier group had received abdominal or pelvic radiotherapy (*P* = 0.666), and 9/105 (17%) and 8/104 (17%), respectively, had received chemotherapy (*P* = 0.958). Intraoperative parameters were comparable between groups (Table 2), although adhesiolysis (*P* = 0.073) and manual anastomosis (*P* = 0.034) were performed more frequently in the HA/CMC powder group.

**Table 1:** **Patient characteristics and medical history at baseline (intent-to-treat population)**

	HA/CMC powder (n = 105)	No adhesion barrier (n = 104)	***P***value
Age in years, mean ± SD	57.6 ± 16.3	56.1 ± 16.5	0.531
Men, n (%)	53 (50.5)	51 (49.0)	0.835
BMI in kg/m^2^, mean ± SD	24.7 ± 4.0	24.3 ± 4.2	0.451
Smoking history, n (%)			0.676
Current smoker	16 (15.8)	18 (17.8)	
Ex-smoker	19 (18.8)	23 (22.8)	
Non-smoker	66 (65.3)	60 (59.4)	
Previous abdominal/pelvic surgery, n (%)	58 (55.2)	61 (58.7)	0.618
Diabetes, n (%)	10 (9.6)	5 (4.8)	0.180
Pre-operative diagnosis, n (%)			0.566
Cancer	48 (45.7)	44 (42.3)	
Diverticulosis	27 (25.7)	36 (34.6)	
Crohn’s disease	11 (10.5)	7 (6.7)	
Ulcerative colitis	5 (4.8)	7 (6.7)	
Polyp (no cancer)	5 (4.8)	5 (4.8)	
Other	5 (4.8)	1 (1.0)	
Endometriosis	2 (1.9)	3 (2.9)	
Polyposis	2 (1.9)	1 (1.0)	
ASA Physical Status classification, n (%)			0.537
P1	37 (35.2)	41 (39.4)	
P2	60 (57.1)	52 (50.0)	
P3	8 (7.6)	11 (10.6)	
Concomitant diseases (≥20% patients in either group), n (%)			
Gastrointestinal, hepatic	46 (43.8)	39 (37.5)	0.353
Cardiovascular	31 (29.5)	35 (33.7)	0.521
Metabolic, endocrine, nutritional	38 (36.2)	26 (25.0)	0.079
Allergic	18 (17.1)	31 (29.8)	0.031
Concomitant medications (≥10% patients in either group), n (%)			
Heparin	51 (48.6)	47 (45.2)	ND
Combination of penicillins*	17 (16.2)	9 (8.7)	ND

*Including β-lactamase inhibitors.

ASA, American Society of Anesthesiologists; BMI, Body mass index; ND, Not determined.

**Table 2:** **Intra**-**operative parameters (intent-to-treat population)**

	HA/CMC powder (n = 105)	No adhesion barrier (n = 104)
Adhesiolysis, n (%)	36 (34.3)	24 (23.1)
Type of resection, n (%)		
Sigmoidectomy and/or left colectomy	44 (41.9)	50 (48.1)
Proctectomy	21 (20.0)	25 (24.0)
Right ileocolectomy	31 (29.5)	20 (19.2)
Total proctocolectomy	5 (4.8)	3 (2.9)
Abdominoperineal amputation	2 (1.9)	2 (1.9)
Total colectomy	1 (1.0)	3 (2.9)
Sigmoidectomy and right ileocolectomy	0	1 (1.0)
Left colectomy and small intestine resection (ileum)	1 (1.0)	0
Anastomosis, n (%)	102 (97.1)	99 (95.2)
Manual	28 (27.5)*	15 (15.2)
Mechanical	74 (72.5)	84 (84.8)
Stomy, n (%)	27 (25.7)	23 (22.1)
Partial omentectomy, n (%)	12 (11.4)	12 (11.5)
Classification of surgical area during surgery, n (%)		
Clean – contaminated	104 (99.0)	104 (100)
Contaminated	1 (1.0)	0 (0)
Exeresis of other organs, n (%)	8 (7.6)	8 (7.7)

*P = 0.034.

There were no differences between the HA/CMC powder and no adhesion barrier groups in perioperative findings (Table 3) except for number of drains used (*P* = 0.021) and mean duration of surgery (*P* = 0.419).

**Table 3:** **Summary of perioperative parameters**

	HA/CMC powder (n = 105)	No adhesion barrier (n = 104)
Estimated blood loss in mL, mean ± SD	116.5 ± 197.7	81.6 ± 95.8
Administration of blood products, n (%)	7 (6.7)	3 (2.9)
Blood sediments in units, mean ± SD	2.0 ± 1.0	1.7 ± 0.6
Perioperative lavage, n (%)	73 (69.5)	73 (70.2)
Intraperitoneal	58 (79.5)	54 (74.0)
Intraluminal	4 (5.5)	2 (2.7)
Intraperitoneal and intraluminal	11 (15.1)	17 (23.3)
Type of intraperitoneal lavage, n (%)		
Localized to intervention site	52 (76.5)	54 (77.1)
Whole cavity	16 (23.5)	16 (22.9)
Use of povidone-iodine antiseptic irrigation, n (%)	19 (18.6)	23 (22.8)
Intraperitoneal	4 (21.1)	5 (21.7)
Intraluminal	12 (63.2)	14 (60.9)
Intraperitoneal and intraluminal	3 (15.8)	4 (17.4)
Drain, n (%)	50 (47.6)	49 (47.1)
Vacuum, n (%)	37 (74.0)	37 (75.5)
Number of drains, mean ± SD	1.1 ± 0.3*	1.6 ± 1.5
Surgery duration in minutes, mean ± SD	216.2 ± 87.1	203.2 ± 81.6
Postoperative oxygen therapy, n (%)	69 (65.7)	56 (53.8)

*P = 0.021.

### Safety outcomes

Adverse events, serious adverse events, and deaths are summarized in Table 4. One patient in each group had an adverse event leading to death: septic shock stemming from aspiration pneumonia in the HA/CMC powder group, and septic shock and multiorgan failure in the no adhesion barrier group; for the patient in the HA/CMC powder group, the investigator assessed the adverse event as not related to treatment.

**Table 4:** **Summary of deaths**, **adverse events, and serious adverse events (events occurring on or after the day of surgery are shown by preferred term)**

	HA/CMC powder (n = 105)	No adhesion barrier (n = 104)
Deaths	1 (1.0)	1 (1.0)
Any adverse event*, n (%)	66 (62.9)^†^	41 (39.4)
Any adverse event considered severe, n (%)^§^	14 (13.3)	5 (4.8)
Most frequently reported adverse events, n (%)^§^		
Hyperthermia	6 (5.7)	3 (2.9)
Incision site abscess	5 (4.8)	3 (2.9)
Pelvic abscess	5 (4.8)	2 (1.9)
Urinary tract infection	5 (4.8)	1 (1.0)
Anastomotic fistula	4 (3.8)	4 (3.8)
Abdominal wall abscess	4 (3.8)	2 (1.9)
Ileus	3 (2.9)	2 (1.9)
Urinary retention	3 (2.9)	1 (1.0)
At least 1 serious adverse event, n (%)	29 (27.6)^†^	11 (10.6)
Any serious adverse event considered severe, n (%)^§^	9 (8.6)	3 (2.9)
Serious adverse events occurring in ≥2 patients in either group, n (%)^§^		
Pelvic abscess	5 (4.8)	2 (1.9)
Abdominal abscess	4 (3.8)	0
Septic shock	1 (1.0)	2 (1.9)
Peritonitis	2 (1.9)	3 (2.9)
Ileus	3 (2.9)	0
Anastomotic fistula	3 (2.9)	4 (3.8)
Gastrointestinal stoma complication	2 (1.9)	0

*All adverse events coded according to the Medical Dictionary for Regulatory Activities version 11.0.

^†^ *P* <0.001 vs. no adhesion barrier group.

^§^Statistical significance not tested.

The overall frequency of adverse events was significantly higher in the HA/CMC powder versus the no adhesion barrier group (66/105 [63%] vs. 41/104 [39%]; *P* <0.001). The overall frequency of serious adverse events was significantly higher in the HA/CMC powder group compared with the no adhesion barrier (*P* <0.001).

Overall, 10/105 (10%) patients in the HA/CMC powder group experienced at least one adverse event that was considered by the investigator as possibly, probably, or definitely related to the investigational product: abdominal pain (n = 2), flatulence (n =2), ileus (n = 1), impaired gastric emptying (n = 1), intestinal obstruction (n = 1), abdominal abscess (n = 2), intestinal abscess (n = 1), incision site abscess (n = 1), anastomotic fistula (n = 1), incision site haemorrhage (n = 1), and postoperative ileus (n = 1). The frequency of treatment-related serious adverse events in the HA/CMC powder group was 4% (4/105 patients), and these were ileus (n = 1) and the SSIs of abdominal abscess (n = 2), intestinal abscess (n = 1), incision site abscess (n = 1), and anastomotic fistula (n = 1). As this was a single-blind study, the relationship to treatment could not be reported in the no adhesion barrier group. There was no relationship between the amount of HA/CMC powder used and incidence of adverse events or serious adverse events (data not shown).

At least one SSI was experienced by 22/105 (21%) of patients in the HA/CMC powder group versus 15/104 (14%) in the no adhesion barrier group (*P* = 0.216), and at least one serious SSI by 13/105 (12%) versus 9/104 (9%), respectively (*P* = 0.38; Table 5). There were no numeric differences between the HA/CMC powder and the no adhesion barrier groups in the most frequently reported serious SSIs of pelvic abscess (4.8% and 1.9%, respectively), anastomotic fistula (2.9% and 3.8%), and peritonitis (1.9% and 2.9%; statistical significance not tested).

**Table 5:** **Overall frequency of SSIs and serious SSIs**, **and listing of all SSIs**

	HA/CMC powder (n = 105)	No adhesion barrier (n = 104)	***P***value
At least 1 SSI, n (%)	22 (21.0)	15 (14.4)	0.216
Deep	13 (12.4)	8 (7.7)	0.260
Incisional	13 (12.4)	7 (6.7)	0.165
At least 1 serious SSI, n (%)	13 (12.4)	9 (8.7)	0.380
Deep	12 (11.4)	8 (7.7)	0.359
Incisional	2 (1.9)	1 (1.0)	1.000
All SSIs, n (%)			
Infections and infestations	20 (19.0)	11 (10.6)	–
Incision site abscess	5 (4.8)	3 (2.9)	–
Pelvic abscess	5 (4.8)	2 (1.9)	–
Abdominal wall abscess	4 (3.8)	2 (1.9)	–
Abdominal abscess	4 (3.8)	0	–
Incision site infection	1 (1.0)	2 (1.9)	–
Abscess intestinal	1 (1.0)	1 (1.0)	–
Abdominal infection	1 (1.0)	0	–
Bacteraemia	1 (1.0)	0	–
Postoperative abscess	1 (1.0)	0	–
Septic shock	0	1 (1.0)	–
Subcutaneous abscess	1 (1.0)	0	–
Injury, poisoning, and procedural complications	6 (5.7)	4 (3.8)	–
Anastomotic fistula	4 (3.8)	4 (3.8)	–
Gastrointestinal anastomotic leak	1 (1.0)	0	–
Incision site complication	1 (1.0)	0	–
Gastrointestinal disorders	2 (1.9)	5 (4.8)	–
Peritonitis	2 (1.9)	3 (2.9)	–
Colonic fistula	0	1 (1.0)	–
Gastrointestinal inflammation	0	1 (1.0)	–

Shown by system organ class and preferred term.

–, Statistical significance not tested.

SSI, Surgical site infection.

In the HA/CMC powder group, the mean ± SD amount of powder applied was 2.7 ± 1.4 g, with 40% of patients receiving 4 to 6 g, 37% receiving 2 to 3 g, and 23% receiving only 1 g. The mean ± SD duration of application was 5.6 ± 3.4 min. The mean ± SD duration of hospitalisation after surgery was 9.7 ± 6.3 days in the HA/CMC powder group compared with 7.5 ± 3.4 days in the no adhesion barrier group (*P* = 0.009).

Adverse events by preoperative diagnosis are summarized in Table 6. A greater frequency of adverse events among patients with cancer was observed than in patients with other diagnoses in both groups; however, the rate of overall adverse events and serious adverse events was higher in the HA/CMC powder group than in the no adhesion barrier group in patients with cancer (33% vs. 17% and 16% vs. 6%, respectively; significance not tested). Similarly, adverse events were more frequent in the HA/CMC powder group versus the no adhesion barrier group in patients with Crohn’s disease and ulcerative colitis (Table 6; significance not tested).

**Table 6:** **Frequency of adverse events**, **serious adverse events, and serious SSIs by preoperative diagnosis**

Preoperative diagnosis	HA/CMC powder (n = 105)	No adhesion barrier (n = 104)
Cancer, n (%)		
Adverse event	35 (33.3)	18 (17.3)
Serious adverse event	17 (16.2)	6 (5.8)
Serious SSI	4 (3.8)	5 (4.8)
Diverticulosis, n (%)		
Adverse event	11 (10.5)	12 (11.5)
Serious adverse event	2 (1.9)	2 (1.9)
Serious SSI	2 (1.9)	2 (1.9)
Crohn’s disease, n (%)		
Adverse event	7 (6.7)	2 (1.9)
Serious adverse event	4 (3.8)	1 (1.0)
Serious SSI	4 (3.8)	1 (1.0)
Ulcerative colitis, n (%)		
Adverse event	5 (4.8)	4 (3.8)
Serious adverse event	3 (2.9)	1 (1.0)
Serious SSI	2 (1.9)	0
Polyp (no cancer), n (%)		
Adverse event	4 (3.8)	4 (3.8)
Serious adverse event	1 (1.0)	1 (1.0)
Serious SSI	0	0
Endometriosis		
Adverse event	1 (1.0)	1 (1.0)
Serious adverse event	1 (1.0)	0
Serious SSI	1 (1.0)	0
Polyposis		
Adverse event	1 (1.0)	0
Serious adverse event	0	0
Serious SSI	0	1 (1.0)
Other		
Adverse event	2 (1.9)	0
Serious adverse event	1 (1.0)	0
Serious SSI	0	0

SSI, Surgical site infection.

Subgroup analysis also indicated a greater frequency of adverse events among patients who had undergone previous abdominal/pelvic surgery than in those who had not in both groups; however, the rate of adverse events and serious adverse events was higher in the HA/CMC powder group than in the no adhesion barrier group, regardless of previous abdominal/pelvic surgery status (data not shown).

### Risk factor analysis

For the serious adverse events risk factor analysis, the covariates included were age, smoking status, smoking frequency, use of corticoids, surgical risk (National Nosocomial Infections Surveillance Index; NNIS), previous cancer, fluorouracil used during the most recent chemotherapy and abdominal or pelvic radiation therapy administration. The probability of a serious adverse event was greater in the HA/CMC powder versus the no adhesion barrier group (OR = 4.08; 95% CI, 1.67–9.95; *P* = 0.002), in younger patients (for age in years, OR = 0.94; 95% CI, 0.91–0.98; *P* = 0.002), and in patients who smoked frequently (OR = 1.06; 95% CI, 1.02–1.10; *P* = 0.006). The probability of a serious adverse event was also greater in patients with a higher level of surgical risk: NNIS index 0 vs. ‒1 (OR = 1.33; 95% CI, 0.53–3.36; *P* = 0.027) and NNIS index 1 vs. ‒1 (OR = 9.87; 95% CI, 1.82–53.53; *P* = 0.027), in those with previous cancer (OR = 3.46; 95% CI, 1.02–11.70; *P* = 0.046) and in those having used fluorouracil during their last chemotherapy (OR = 7.12; 95% CI, 1.52–33.42; *P* = 0.013).

For the deep SSIs risk factor analysis, age and smoking frequency were included as covariates. The probability for a deep SSI was greater in younger patients (for age in years OR = 0.97; 95% CI, 0.94–1.00; *P* = 0.0362) and in patients who frequently smoked (OR = 1.05; 95% CI, 1.01–1.09; *P* = 0.010). No significant effect of treatment was observed (OR = 0.58; 95% CI, 0.22–1.53; *P* = 0.269).

### Ease of use and reliability

**Figure 2:**
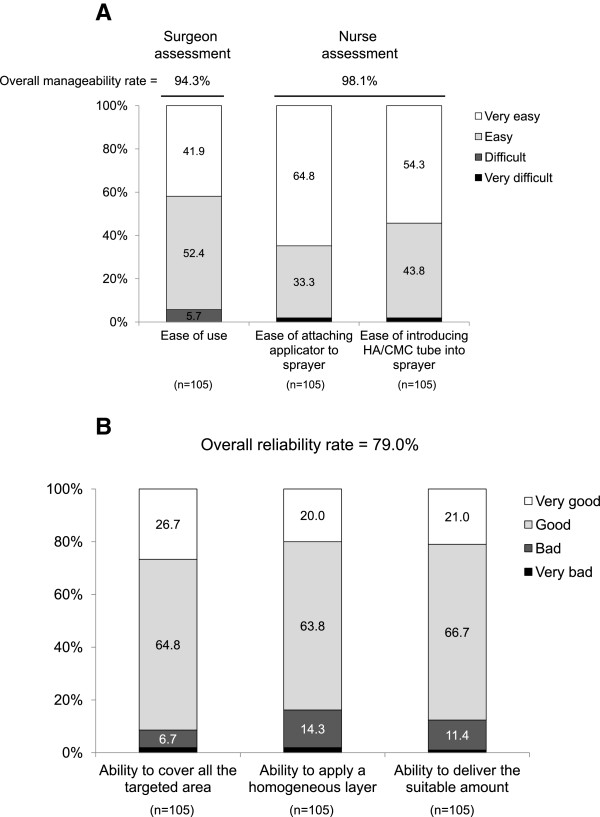
**Ease of use and reliability.** **(A)** Ease of use of HA/CMC powder, as assessed by surgeons and nurses; **(B)** Reliability of HA/CMC powder, as assessed by surgeons. Overall manageability/reliability rates determined by overall number of cases scoring 3 or 4 on a 4-point scale (3 = easy or good, 4 = very easy or very good). For multi-component items, all were required to achieve a score of 3 or 4.

HA/CMC powder was considered to be manageable by nurses in 98% (103/105) of procedures, based on ease of use assessment (Figure 2A). Surgeons considered HA/CMC powder manageable and reliable in 94% (99/105) and 79% (83/105) of procedures, respectively (Figure 2A,B).

## Discussion

In this exploratory study evaluating safety outcomes, there were statistically significant differences between the HA/CMC powder and the no adhesion barrier groups in the frequency of adverse events and serious adverse events (*P* <0.001). The occurrence of abdomino-pelvic abscess was more frequent in the HA/CMC powder group than in the no adhesion barrier group, although there were no statistically significant differences between groups in the frequency of SSIs or serious SSIs. Surgeons considered HA/CMC powder easy to use and reliable, and nurses also considered HA/CMC powder to be manageable in the majority of procedures. Based on the observed safety findings, the use of HA/CMC powder is no longer being pursued in colorectal laparoscopic surgery.

The most frequent types of adverse events were those often encountered in patients undergoing colorectal or intestinal resection (‘infections and infestations’ and ‘gastro-intestinal disorders’). Among the adverse events and serious adverse events that were considered by investigators to be related to treatment, there was not one particular type of event or safety issue that was more frequently reported than others. Frequency appeared to be independent of the quantity of HA/CMC powder applied. There was a trend towards a higher frequency of adverse events in patients with cancer versus other aetiologies, and a higher frequency of adverse events in the HA/CMC powder group versus the no adhesion barrier group in patients with cancer and those with inflammatory pathologies, although the study was not powered to detect a significant difference for such a subgroup analysis. As this was an exploratory study, no sample size calculation was made, but the enrolment of approximately 100 patients per group would have provided a 95% CI of 25.6 to 44.4 for the detected difference, based on an expected overall rate of serious adverse events of 35% [21].

Other studies report that SSIs occur following colorectal surgery with a frequency ranging from 5% to 45% [25–33]; the frequency of SSIs observed in this study falls within this range. The risk factor analysis in our study found no significant effect of treatment on risk for deep SSIs. Factors that appeared to be associated with a greater risk were lower age and frequent smoking. There was a significant treatment effect on risk of serious adverse events; other factors that appeared to be associated with a greater risk were lower age, frequent smoking, high level of surgical risk, previous cancer, and having used fluorouracil during the last chemotherapy. However, given the exploratory design of this study, these results must be considered with caution.

The findings of our study were unexpected given that no safety issues were identified in a pilot study by Fossum et al. assessing the use of HA/CMC powder in laparoscopic myomectomy [20]. In their study, Fossum et al*.* observed no overall difference in adverse event frequency between the HA/CMC powder and the no adhesion barrier groups (67% vs. 60%, respectively), and only one patient experienced a serious adverse event (leukaemia, in the no adhesion barrier group). No adverse events directly related to HA/CMC powder were identified, as determined by the surgeon, and there were no reports of SSIs or intra-abdominal abscess. Furthermore, in animal models, HA/CMC powder has been shown to be effective in preventing adhesions without affecting wound healing [18, 19]. In a rat model of anastomotic healing, there were no statistically significant differences between the HA/CMC powder and the no adhesion barrier groups in number of deaths or short-term complications of abscess formation, bowel obstruction, proximal colonic dilatation, and wound dehiscence [19].

In the present study, an important consideration was that the patient population enrolled had a high level of comorbidity and a high probability of postoperative morbidity. This population was intended to be heterogeneous, comprising a broad range of patients undergoing various abdominal surgeries for a variety of diagnoses, including cancer. The risk of postoperative infection is high in patients undergoing gastrointestinal surgery as a result of opening the bowel, whereas gynaecologic surgery is usually a ‘clean’ (Class I) wound in comparison [34]. Although direct comparison with other studies is not possible, the overall rate of adverse events in the no adhesion barrier group in this study (39%) was similar to overall postoperative comorbidity rates in prospective studies of open or laparoscopic colorectal surgery (~25% to 35%) [21, 35]. However, this does not explain the significantly higher frequency of adverse events and serious adverse events in the HA/CMC powder group versus the no adhesion barrier group. Intraoperative and perioperative parameters that differed between groups were adhesiolysis and manual anastomosis (both performed more frequently in the HA/CMC powder group), number of drains (lower in the HA/CMC powder group), and mean duration of surgery (longer in the HA/CMC powder group).

As the safety of the film formulation has been confirmed in clinical studies of gynaecological and abdominal surgery [13, 14, 36, 37], the powder formulation of HA/CMC used in this study is likely to be an important contributing factor for the increased frequency of adverse events and serious adverse events. One study noted that wrapping a new bowel anastomosis with HA/CMC film adhesion barrier increased the risk of anastomotic leak and related events such as fistula, peritonitis, abscess, and sepsis [24], possibly by interfering with anastomosis healing; this practice is therefore contraindicated. For adhesion barriers with a liquid or gel formulation, greater diffusion of product across the peritoneal surface may play a role in increasing adverse event risk as a result of application occurring away from the wound site. Gels may also have a propensity to pool away from the wound site. A study of 0.5% ferric hyaluronate gel adhesion barrier in open colorectal surgery was suspended owing to significantly greater morbidity versus the control group (distilled water, 65% vs. 27%; *P* = 0.031). There was a higher rate of anastomotic dehiscence (5/17 vs. 1/15, respectively; not significant) [34] but investigators were unable to determine whether anastomotic healing was disrupted directly by the adhesion barrier or by infection associated with gel use. Furthermore, icodextrin 4% fluid is indicated for use only in patients undergoing gynaecological laparoscopic adhesiolysis in the USA owing to occurrence of serious complications following laparotomy and bowel resection/repair [38]. Pre-clinical data suggested that HA/CMC powder demonstrated more rapid dissolution and wider diffusion than HA/CMC film [19], with a greater capacity for fluid absorption and release of a higher concentration of polymer into solution within the first few hours of hydration than the equivalent amount of HA/CMC film [18]. As application of HA/CMC film to anastomoses is contraindicated owing to the potential for an increase in anastomotic leak-related events [24], great care was taken in our study to practise avoidance of anastomoses with HA/CMC powder. Nevertheless, due to the potential for greater diffusion of the powder formulation, the authors speculate that migration away from the application site to anastomoses could have occurred in some cases. Furthermore, over-hydration of the HA/CMC powder might have resulted in pooling of the resulting gel away from the application site, raising the possibility of migration onto an anastomosis or provision of a nidus for abscess; such migration to anastomoses might increase the rate of SSIs.

There is no current evidence of a biological effect of HA/CMC [39–41], although hyaluronan is known to promote cell proliferation and migration [42]. Histological analysis of tissues from pre-clinical studies indicated that macrophage response and rate of remesothelialisation appeared to be similar for both film and powder formulations [18]. There may be potential for powder and gel formulations to act as sites of origin for abscess formation, and/or to be associated with septic complications [19]. An animal study assessing the effect of adhesion barriers on the progression of bacterial infection in the peritoneum found that HA/CMC films had no effect when compared with control groups (saline), whereas some modified gel formulations appeared to increase mortality [43]. Furthermore, testing of HA/CMC powder in a rat model of sepsis involving simultaneous exposure to variable doses of *Escherichia coli* and sterile caecal contents identified a safety signal on repeat testing (unpublished data, Genzyme Biosurgery), suggesting adverse effects in the presence of active infection or gross caecal contamination. Pre-clinical studies observed an infection potentiation resulting from a physical interaction of HA/CMC powder with caecal material and bacteria during the initial stages of hydration within the first 4 h of implantation (unpublished data, Genzyme Biosurgery). Although our study excluded patients with pathology requiring class IV contamination surgery and those with abdominal abscesses and/or peritonitis or abdominal cavity infection, it is possible that in some patients the presence of HA/CMC powder in combination with luminal contents resulted in adverse events owing to peritoneal bacterial contamination.

The main limitation of this trial is its exploratory nature and lack of efficacy assessment. The study population enrolled was a heterogeneous and difficult-to-treat population with a high risk of morbidity and a broad range of aetiologies; therefore, these results cannot be extrapolated to other patient populations. The key strength of the study was the care taken to standardize the application technique according to the instructions provided. The findings suggest that the observed safety signals are most likely related to the specific formulation (powder); therefore, these results should not be extrapolated to other formulations of adhesion barriers.

## Conclusions

This exploratory study found significantly higher rates of adverse events and serious adverse events in the HA/CMC powder group compared with the no adhesion barrier group, indicating a global safety signal in the laparoscopic application of HA/CMC powder in colorectal and small bowel resection. Thus, further development of HA/CMC powder is no longer being pursued for use in patients undergoing colorectal and small bowel laparoscopic surgery, given that this population has an elevated risk for postoperative comorbidity.

## Abbreviations

CI: Confidence interval
HA/CMC: Hyaluronic acid/carboxymethylcellulose
NNIS: National Nosocomial Infections Surveillance Index
OR: Odds ratio
SSIs: Surgical site infections.
